# The Molecular Floodgates of Stress-Induced Senescence Reveal Translation, Signalling and Protein Activity Central to the Post-Mortem Proteome

**DOI:** 10.3390/ijms21176422

**Published:** 2020-09-03

**Authors:** Valerie C. Wasinger, Darren Curnoe, Ceridwen Boel, Naomi Machin, Hsiao Mei Goh

**Affiliations:** 1 Bioanalytical Mass Spectrometry Facility, Mark Wainwright Analytical Centre, University of New South Wales Sydney, Kensington, NSW 2052, Australia; 2 Palaeontology, Geobiology and Earth Archives Research Centre, University of New South Wales Sydney, Kensington, NSW 2052, Australia; ceridwen@uow.edu.au (C.B.); n.machin@unsw.edu.au (N.M.); hsiao.goh@unsw.edu.au (H.M.G.); 3 ARC Centre of Excellence for Australian Biodiversity and Heritage, University of New South Wales Sydney, Kensington, NSW 2052, Australia; 4 Centre for Global Archaeological Research, University Sains Malaysia, Penang 11800, Malaysia

**Keywords:** death, coordinated gene-expression, ribosomal binding proteins, paleoproteomics, post-mortem, senescence, mitochondrial dysfunction, inflammation

## Abstract

The transitioning of cells during the systemic demise of an organism is poorly understood. Here, we present evidence that organismal death is accompanied by a common and sequential molecular flood of stress-induced events that propagate the senescence phenotype, and this phenotype is preserved in the proteome after death. We demonstrate activation of “death” pathways involvement in diseases of ageing, with biochemical mechanisms mapping onto neurological damage, embryonic development, the inflammatory response, cardiac disease and ultimately cancer with increased significance. There is sufficient bioavailability of the building blocks required to support the continued translation, energy, and functional catalytic activity of proteins. Significant abundance changes occur in 1258 proteins across 1 to 720 h post-mortem of the 12-week-old mouse mandible. Protein abundance increases concord with enzyme activity, while mitochondrial dysfunction is evident with metabolic reprogramming. This study reveals differences in protein abundances which are akin to states of stress-induced premature senescence (SIPS). The control of these pathways is significant for a large number of biological scenarios. Understanding how these pathways function during the process of cellular death holds promise in generating novel solutions capable of overcoming disease complications, maintaining organ transplant viability and could influence the findings of proteomics through “deep-time” of individuals with no historically recorded cause of death.

## 1. Introduction

The leading contributors to death for older Australians are vascular and coronary heart disease, dementia related disorders, infection and immunological complications [1]; common causes to the decline in health echoed around the world. However, while these causes of death can be statistically represented by these all too familiar descriptors, it is the underlying ageing of biochemical mechanisms that ultimately contributes to our demise. It has been widely assumed that death rapidly causes a complete shutdown of all molecular activities including transcription, translation, protein modifications and signalling cascades. This is definitely not the case [2,3,4,5], however, with the post-mortem proteome representing an intriguing map of the potential of a biological system. In living organisms, the decommissioning of cells occurs due to precisely expressed genetic and epigenetic controls that reduce the risk of the accumulation of damaged cells [6]. In death, control at a genetic and epigenetic level extends significantly through post-mortem (PM) time in an environment that is increasingly managed at the protein level to affect cellular shutdown. The understanding of this shutdown process is in its infancy. Greater awareness of the activation of “death” pathways have particular relevance to the study of many diseases related to ageing and could influence the findings of proteomics through “deep-time” of individuals with no historically recorded cause of death.

The highly conserved processes of cellular turnover are heritable and also recruited for the purposes of organismal development, maintaining homeostasis and immune function in living organisms [7]. Presumably in death, the role of driving cellular wellbeing is maintained for as long as possible. In this way parallels can be drawn by the analysis of cells under duress to describe the pathways triggered by organismal death. The processes of cell death can involve autophagy, necrosis, apoptosis, anoikis, ferroptosis, oncosis (ischemic cell death) and pyroptosis (innate immune activation form of cell death) [8]. These pathways also involve specific proteins including caspases, calpains, cathepsin, nucleases, transglutaminases and kinases [9]. These proteins make up the functional effectors of the cell and as such, their involvement in post-mortem time and the downstream effect of their activity, is crucial for understanding the role proteomics can play in determining disease progression; this includes the prospect of involvement of these processes in neurodegeneration, cancer, diabetes mellitus, cardiovascular, autoimmune diseases and the viability of organs for transplantation.

Parallel genome-scale measurements of mRNA abundance, half-life and corresponding protein levels have demonstrated that protein abundance and mRNA abundance is correlated [10]. However, changes in translation efficiency rather than transcriptional control can account for protein abundance differences [10,11]; and an uncoupling of transcription and translation control can occur during cellular stress [12]. Gene-expression has been shown to continue well after death [3,4,13], with increases in some transcripts at their maximum 48 h PM [13], in addition to functional enrichment of transcripts associated with inflammation, stress, transport, cancer and development [13]. Other genes and proteins have also been determined to increase at differing time points PM and these have been used in calculations to determine the PM interval in forensic investigations [2]. Transcriptomic studies have demonstrated a structured shutdown of transcription to 96 h PM with a gradual loss of regulatory networks [14]. While gene expression is fundamentally under stochastic control [15], simultaneous protein synthesis using coordinated mRNA translation through post-transcriptional control is another mechanism to efficiently maintain protein networks and larger macromolecules.

Protein synthesis carries a high cellular energy demand. Consumption of ATP and reduction of the glycogen stores is inevitable across PM time, but is also simulated during periods of cellular stress. During stress, synthesis is conservative while RNA binding proteins (RBP) reprogram translation post-transcriptionally to favour synthesis of repair proteins. They do this by recognising upstream (un)translated regions of their mRNAs and compete with microRNAs and non-coding RNAs for their binding sites [16], including in the PM transcriptome [3]. As much as 90% of cellular stress response mRNAs are being controlled through the action of RBPs in conjunction with post-translational modifications to support genomic stability and integrity in an energy efficient manner [17]. It can be intuited that there should be a dominance in death for translational regulation given the high energy cost associated with protein synthesis from the transcriptional level. All forms of RNA transcripts are controlled by these regulatory RNA binding elements, influencing the levels of genes and pseudogenes and expediting the stress response to modulate the expression profile. This has implications in pathological conditions such as cancer [14,18,19], particularly in pro-survival proteins [20].

The translation of mRNAs by non-canonical approaches can have an immediate effect on the microenvironment of individual cells [21,22]. For instance, different cell types are able to harness pro-survival strategies through the modification of protein synthesis machinery via the unfolded protein response (UPR). The α subunit of eukaryotic initiation factor 2 (eIF2α) mediates translational regulation in response to stress in a GTP dependent manner; a scenario which is also mimicked in the PM transcriptome [13]. These processes are heavily regulated by phosphorylation within the protein complex and translational repression of most mRNA with a selective upregulation of around 2.5% of total mRNAs [23]. The consequence of this allows conservation of resources in the selective drive for cytoprotective transcription, cellular adaptation, but leads to eventual apoptosis. Protein folding is an energy demanding process that requires glycosylation of proteins and the reduction of ATP to effect calcium accumulation in the endoplasmic reticulum promoting chaperonin activity. Accumulation of unfolded proteins can result from disrupted calcium transfer, nutrient deprivation, hypoxia and reactive oxygen species (ROS). This triggers the evolutionarily conserved stress response pathways including the (UPR) and DNA damage response (DDR).

Cellular energy production occurs in two ways. One of the major ATP-producing pathways is glycolysis which occurs in the cytoplasm of almost all cells without the requirement for oxygen [24,25]. However, in the absence of oxygen, pyruvate cannot be completely oxidised to carbon dioxide resulting in intermediate products such as lactic acid. When oxygen is available the pyruvate produced by glycolysis to generate two additional ATP and two NADH molecules is converted to acetyl CoA or oxaloacetate which is itself a 2-carbon energy carrier. In functioning mitochondria, acetyl CoA is then pushed through the tri carboxylic acid (TCA) cycle to generate three more NADH molecules and two other carrier molecules FADH_2_ and GTP [24,25]. The 2nd major process for energy production, called oxidative phosphorylation, involves the electron transport chain within the inner membrane of a mitochondrion. It involves the transfer of electrons from NADH and FADH_2_ through several protein complexes and ultimately to oxygen to form water [25]. The cycling of electrons across these complexes causes an extrusion of H ions. Cells are able to harness this gradient to create three additional ATP molecules. Oxidative phosphorylation creates 15 times more ATP energy than fermentation. Other energy stores such as sugars (glycogen) and lipids are able to be held in reserve and are likely to provide the cell with ~24 h to several weeks’ worth of energy respectively [25]. Cell death is tightly associated with oxidative stress which results in peroxidation of lipids and produces reactive oxygen species (ROS). Together, glycolysis and oxidative phosphorylation coordinate energy based on the cellular demand, growth state and microenvironment of a cell. When there is a lack of oxygen, such as is present internally in dense populations of tumour cells, there is a drive toward aerobic glycolysis with higher glucose transport and production of lactic acid in a process called the Warburg effect [26,27]. Reduction of pyruvate to lactate regenerates NAD^+^ so that glycolysis can proceed and is a dead end in metabolism. The alternative use of glucagon and lipids via β-oxidation is not as efficient and also requires oxygen to generate energy.

The maintenance of these pathways in life is significant for a large number of biological scenarios. However, an in depth understanding of how these pathways function during the process of cellular death holds even more promise in generating novel solutions capable of overcoming disease complications. Proteomics is able to address the interplay of all affected pathways concurrently. Here we report the use of high-resolution, mass spectrometry to retrieve proteomic profiles of the mouse mandible through 720 h of PM time. We assess whether the effectors of the cells (the proteins) are able to function and regulate in a coordinated and sequential manner beyond death and determine changes in pathways through PM time. To confirm enzymes remain functional and active, we have measured the activity of phosphoglucomutase (PGM). This is the first study to consider the changes in global protein abundances and their contribution to variations in associated networks across PM time. The protein profiles are remarkably similar to the stress-induced premature senescence (SIPS) phenotype. This study goes beyond the limits of survival to demonstrate that the machinery for survival persists and continues to function.

## 2. Results

The mass spectrometry proteomics data have been deposited to the ProteomeXchange Consortium via the PRIDE [28] partner repository with the dataset identifier PXD016477. The database (Spectral count through Post-Mortem Time.xls) has the option to filter for specific protein properties thus allowing the tracking of proteins relevant in other contexts such as disease, ageing and paleoproteomics.

The effect of protein loss over time was ameliorated by normalising LCMS experimental data by a scalar factor for each sample. This adjustment factor averaged 1.3 for all samples, indicating that a fold change of >1.3 would indicate fold change significance. We also observed increase and decrease of protein abundances for the same proteins across the time points consistently in numerous mice and have highlighted the consistency of abundance of collagen peptides across PM time (Figure S1, red lines). Collagen is a common protein within mouse dentaries that does not undergo degradation in the time scales assayed [29], thus it can be used as a sampling control. An alternative approach is to demonstrate this using an antibody-based method (concordance of MS and antibody immunoblot data are presented in Figure S1 for collagen and confirms the suitability of using ColA2 as a sampling control. As additional measures, we have reported the observed intensities as 1 h-time point normalised values for observations present in multiple mice, while missing values have been reported as not detected (ND).

**Figure 1:**
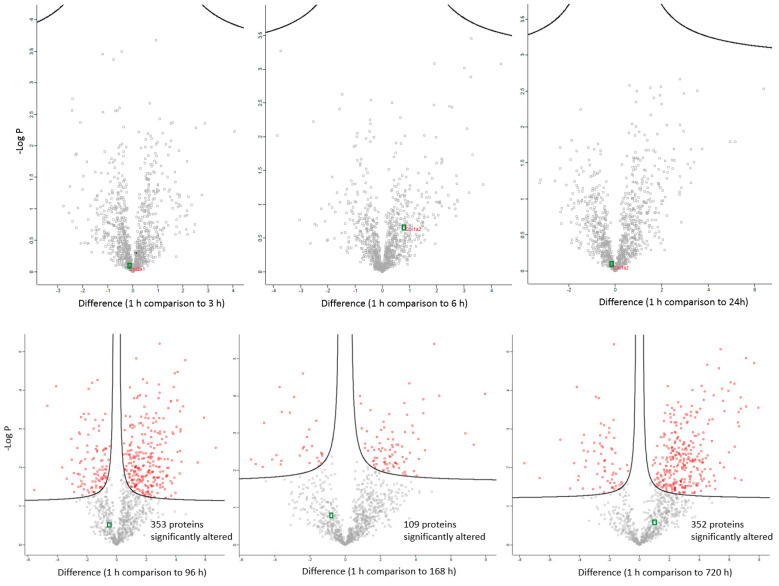
Differentially abundant proteins across PM (post mortem) time scales. Significantly differential proteins are demarcated by red points. Collagen is demarcated by green points. Significantly altered abundances occur after 24 h post mortem compared to the 1 h time point with up to 352 proteins changing in their abundance 1 month after death (720 h).

**Figure 2:**
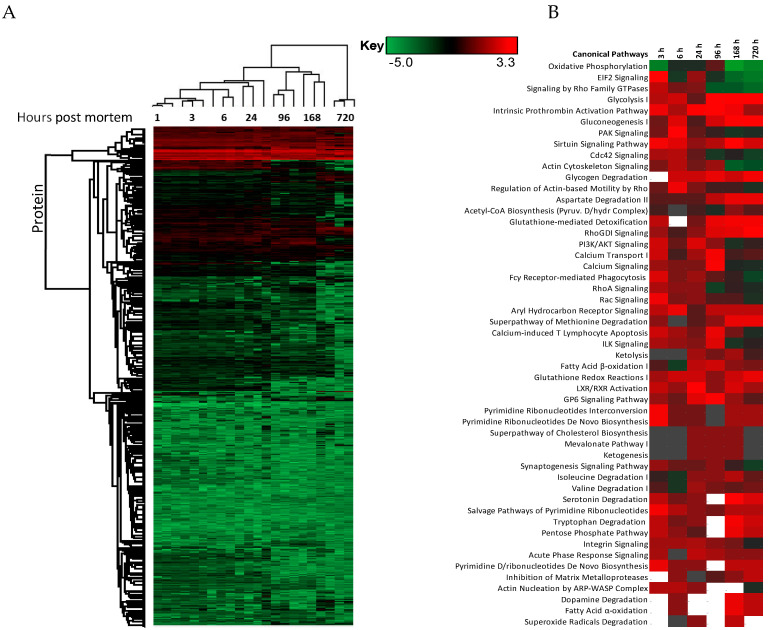

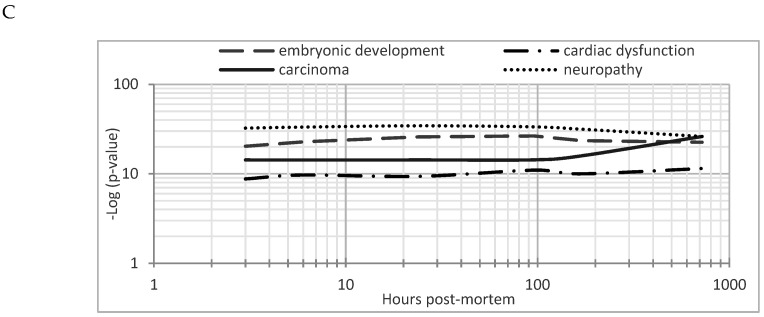
Hierarchical clustering using z-scores of protein abundance across PM time scales. (**A**) Hierarchical clustering based on Z-score normalised log2-transformed intensities show the biological replicates of each PM time point cluster together. The greatest difference was observed between 24 to 96 h and 720 h PM. Increases in protein abundance levels (red) and decreases (green) can be demonstrated across PM time with black indicating no change. Groups of proteins are seen to modulate together. These correlate to (**B**) enriched canonical protein networks and are better visualised at the functional level based on changes in z-scores normalised to 1 h-time points. The corresponding enriched pathologies are provided in (**C**) based on *p*-values of enriched protein pathways triggered through PM time. These pathologies are commonly observed in the study of many living systems.

The changes in expression were followed for 1258 proteins across the PM proteome to 720 h PM (2 peptide identification and FDR < 0.01). Within this dataset 133 proteins associate with positive transcriptional control or act as transcriptional factors, 148 proteins have molecular functions involving RNA binding, while the function of 30 proteins are related to RNA trafficking, more than 30 proteins are involved in translation, 18 proteins are involved in elongation of the polypeptide chain and 28 proteins belong to glycolysis and TCA cycle pathways. Volcano plots show the change in abundance significance across PM time, normalised to the 1 h time-point with significantly different protein abundances demarcated by red in Figure 1. The majority of protein abundances remain insignificantly modulated, most apparent in 3, 6 and 24 h PM. From 24 h, many proteins modulate in abundance with late PM time scales demonstrating major changes in protein abundance. These modulations can be visualised using hierarchical clustering in Figure 2. The majority of proteins are seen to reduce in abundance as might be intuitively predicted to result from death. Of interest is the gradual decrease in groups of proteins (green) and the increase of proteins (red) through PM time (Figure 2A). Pathway enrichment studies (Figure 2B) align well with the hierarchical clustering analysis to demonstrate modulated protein abundances related to energy metabolism (glycolysis/gluconeogenesis/TCA), signalling (RhoGDI/AKT/sirtuin/calcium), and detox pathways (ethanol-/glycogen-/methionine-degradation). Decreases in actin cytoskeletal signalling and oxidative phosphorylation were also observed. Sequential protein abundance modulations are also apparent in LXR activation maximum (red) at 24 h, calcium transport/signalling maximum at 96 h, fatty acid β-oxidation maximum at 96 h and fatty acid α-oxidation at 168 h. Clustering demonstrates the “relatedness” of 1–24 h PM data and divergence of 96–720 h PM data. These differences reflect the chemical and biological activity of proteins within the dentine crystalline matrix through PM time. At the pathway level, there is a decrease (green) in oxidative phosphorylation. Increases (red) in glycolysis and gluconeogenesis and G6P signalling, RhoGDI signalling, pentose phosphate pathway and fatty acid alpha/beta oxidation are also observed. Protein maxima were observed for EIF2 signalling at 3 h; prothrombin activation pathway at 24 h; calcium transport, signalling and calcium induced T-lymphocyte apoptosis at 96 h; and sirtuin signalling at 168 h. Although, we observed that canonical pathways of protein synthesis and translation did not change significantly through PM time with z-score maxima of 0.8 and 0.9, respectively (equivalency is represented by z-scores of between 1 and −1 and are insignificant, Figure S2), the finding that translational and protein synthesis machinery prevails, and that some proteins representative of these pathways are increasing in abundance would suggest that these processes play a functional role in the PM proteome. Proteins describing known pathologies were enriched in the PM proteome (Figure 2C). We observed increases in inflammatory process, cancer, development of neuropathies and tissue/embryonic development early in PM time. Increased significance was observed overall for carcinoma Figure 2C.

**Figure 3:**
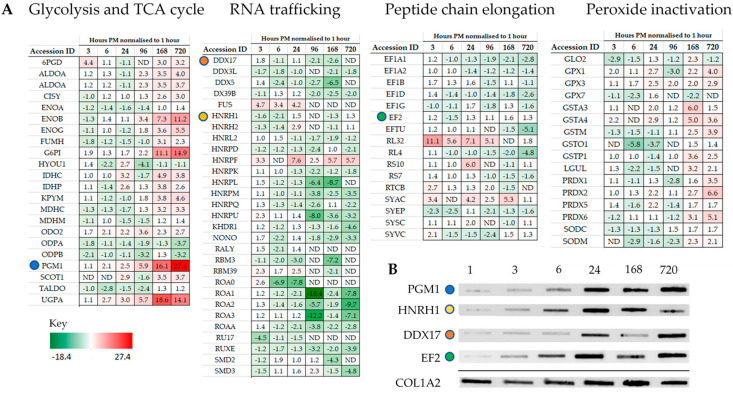
Representation from major pathways can be followed through PM time with some proteins increasing in abundance as far as 720 h PM. (**A**) Proteins representative of glycolysis and TCA cycle pathway, RNA trafficking, and elongation of the polypeptide change. (**B**) Protein abundance by antibody detection can be compared for the same mice against the sampling control protein Col1A2. Coloured circles highlight proteins assayed across both mass-spectrometry and antibody detected datasets.

**Figure 4:**
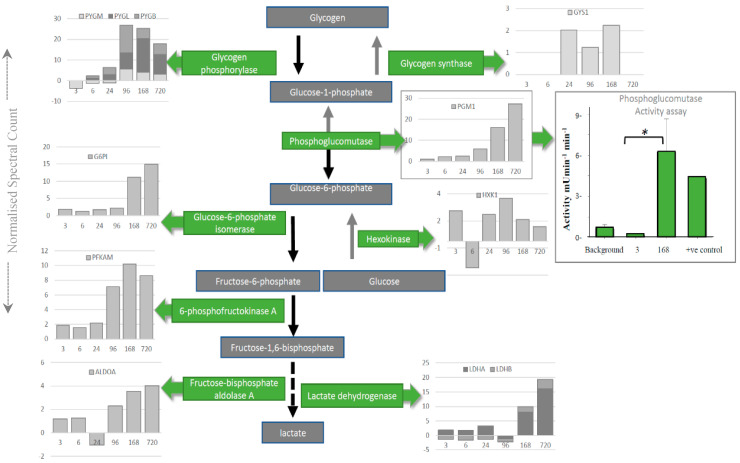
Depletion of glycogen by the glycolytic enzymes is mapped using spectral counting through PM time scales. The enzyme activity was assayed for phosphoglucomutase (PGM) to demonstrate that increased enzyme activity (green bars on boxed PGM graph) corresponds to the increase in protein abundance (grey bars on boxed PGM graph) late into PM time scales. * means *p* < 0.05.

Intriguingly, individual proteins within the major pathways of the glycolysis and the TCA cycle, trafficking of RNA, translation (Figure 3), management of oxidative stress and representation from the ubiquitin proteasome (Tables S1–S3), can be followed across PM time. Increases are seen in pathways involved in alternative forms of energy processing (Table S4), as well as glycolysis/gluconeogenesis at critical points in the pathway (Figure 3A and Figure 4). A vital protein in this pathway, phosphoglucomutase (PGM), was observed to increase in abundance sequentially through PM time. This multi-directional enzyme interconverts glucose-1-phosphate and glucose-6-phosphate in a process that generates NADH. In addition to spectral counting and antibody detection of protein abundance, measurement of PGM enzyme activity demonstrated a functional and active enzyme at 168 h PM (Figure 4). All other enzymes in this pathway have also been accounted for and each display maxima at similar time points through PM time. A temporal association of increasing abundance would suggest the entire pathway is functional and active after death. TCA cycle proteins, isocitrate dehydrogenase (IDH) and malate dehydrogenase (MDH) were also present; while RNA binding proteins important to the post-transcriptional regulation of proteins are observed to increase to 24 h PM. Still detected after 168 h PM was KH domain-containing RNA binding protein1 (KHDR). These findings are also correlated by antibody detection of four proteins observed to increase through PM time (Figure 3B).

**Figure 5:**
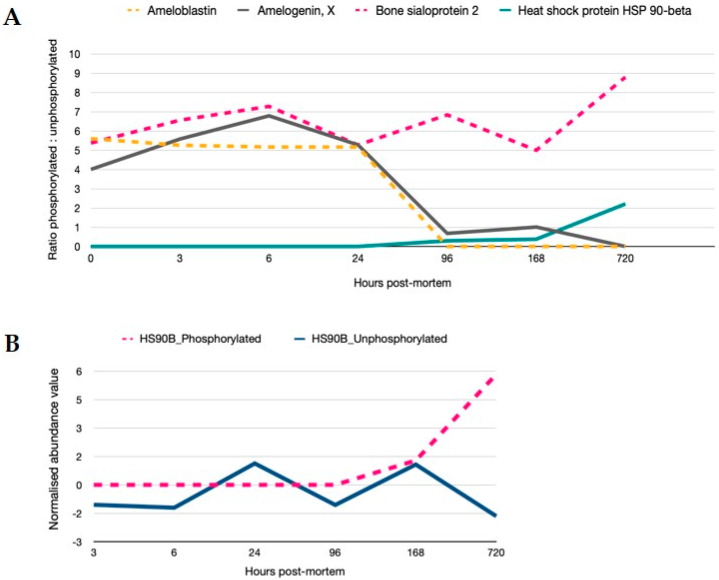
Regulation of signalling cascades are still active in PM time. (**A**) Ratios of phosphorylated to unphosphorylated versions of the proteins are provided (>2-fold variation in phosphorylation). In particular, proteins bound within the crystalline matrix and inherent to the mandible structure such as ameloblastin, amelogenin, bone sialoprotein and HSP90 demonstrate sequential changes in phosphorylated states; steady state levels of phosphorylated sialoprotein, a decrease in phosphorylation from 24 h for amelogenin and ameloblastin; and an increase in the phosphorylated HSP90 beta protein after 96 h PM. (**B**) Relative quantitation of phosphorylated and unphosphorylated HSP90 normalised to 1 h time point show an uncoupling of function from 168 h.

Signalling is controlled by post-translational regulation of proteins. Phosphorylation of serine and threonine residues is reflective of signalling cascade regulation and was present for 30 proteins in the data set. Proteins demonstrating >2-fold modulation in phosphorylation are shown in Figure 5. Proteins that are constitutively phosphorylated remain so as evidenced by the ratio of phosphorylated to unphosphorylated protein for bone sialoprotein, while for ameloblastin and amelogenin, phosphorylation is reduced from 24 h. Phosphorylation on proteins such as heat-shock 90- become hyperabundant once the regulation of its constitutively controlled unphosphorylated state is relaxed. MS spectra of phosphorylated peptides are provided in the supplementary information (Figure S3).

## 3. Discussion

### 3.1. Relationship to Ageing and Senescence

Understanding the mechanisms driving ageing is often observed from the standpoint of a disease at the level of a protein or transcript. In this manner, many studies have demonstrated derangement in energy metabolism, changes to protein synthesis, cellular senescence and chronic inflammation [30,31,32,33]. However, most of these studies fail to integrate these pathways collectively to observe the repercussions on cells at the level of an entire organism’s “health”. This is because the complexity of these phenotypes can be associated both with pathological progression as well as be used advantageously for regeneration in the clinical setting depending on the context of the microenvironment [34]. This has significant implications in the clinic for organ transplant recipients with studies demonstrating the increased risk of inflammation, cardiovascular risk, rejection and development of cancer [35]. Colorectal, renal, leukaemia and melanomas are amongst the extensive list of malignancies diagnosed within a few years of transplantation [36]. For example, one of the most common malignancies following transplantation is Kaposi sarcoma which has a standardized incidence ratio (SIR) averaging 208, a significant increase when compared to 20 SIR in a pre-dialysis study population [36]. In recent years, the power of proteomics has shown how large scale data can inform on senescence associated phenotypes and ageing [30,37,38]. Here we applied the power of temporal proteomics to demonstrate that pathways enriched in the death proteome are the same pathways triggered in phenotypes of ageing and senescence which will have sobering consequences for all our well being.

Proteins are the functional components of the cell and have average half-lives nine times longer than mRNA [11]. Uncoupling the transcript/protein abundance equivalency under cellular duress is inevitable given the energy expenditure required for transcriptional protein synthesis to occur. Constitutive cellular processes such as translation and energy production typically involve both stable mRNA and stable proteins [11]. Proteins with these properties are dominated by the ribosomal proteins as well as those involved in glycolysis and the TCA cycle. As these pathways provide the cell with the continued ability to renew proteins as well as energy, this stability can extend their functionality well into PM time within the protective crystalline matrix of the mandible. Others have also shown that proteins co-existing in multiprotein complexes, or that have a larger number of transcripts, have a turn-over rate extending to 20 h [39]. These scenarios fit well and are mirrored in this mouse study by the identification of 78 ribosomal proteins (~6% of all proteins) for time scales of 1–24 h post-mortem, but falls short of satisfying the increase in protein abundances observed beyond 24 h. The control of protein abundance by translational feedback systems can simultaneously regulate the expression of multiple proteins from existing mRNA allowing control over the coordinated expression of protein networks [40]. RBP which are able to bind message and enable promiscuous gene and pseudo gene expression have been used to explain mRNA maxima as far out as 96 h PM [13]. In this study, 10 RNA binding proteins were identified in the dataset with high but decreasing abundance in all mice extending their activity as far as 168 h PM. The RBP, KHDR1, a known regulator of mRNA stability and alternative splice site selection [41], was detectable in two of three mice at 720 h. In addition, the catalytic machinery to enable the addition of amino acids onto growing polypeptide chains in association with ribosomal proteins were identified. The increases in expression observed using immunoblotting for DDX17 an RNA helicase involved in the unwinding of RNA secondary structure required for translation, as well as EF2 required for translocation of tRNA and mRNA down the ribosome are almost equivalent to the relative abundances observed using spectral counting. Seven tRNA ligases were identified with five remaining detectable at 720 h PM despite significant reduction in oxidative respiration. In this study, spectral counting as well as antibody detection demonstrate that the machinery for protein synthesis is present.

### 3.2. The Stress Response

Augmented aerobic glycolysis due to death results in higher glucose utilisation. Hypoxia forces the cell into a higher glycolysis state and other forms of energy production to maintain homeostasis, a result analogous to the Warburg effect. The hypoxia up-regulated protein HYOU1 is a member of the heat-shock protein 70 family with alternative transcription and translation sites and a cis-acting segment involved in stress-dependent induction, a known feature used by the PM transcriptome to increase abundance in death [3]. Expression of this gene suppresses apoptosis and was found to dramatically increase with a maximum at 24 h PM, followed by its reduction, but still detected in at least two of three mice 720 h after death. Phosphoglucomutase (PGM) represents a critical metabolic switch to the immortalisation of cancer cells under hypoxic conditions. Once competed for resources become low, the deprivation of glucose reverses glycolysis and consequently elevates the enzyme PGM as well as the enzymes at each step in the catabolic conversion of glycogen to glucose and ultimately to lactate and ROS. Aberrations in the abundance of enzymes involved in glycogen metabolism, particularly during glucose deprivation, have been noted for a number of cancers [42,43] and are associated with elevated levels of PGM, premature senescence [44], cancer cell survival and poor prognosis [42]. Elevation of the PGM protein with increased activity of the PGM late into PM time, along with the increase in other glycolytic enzymes, demonstrates a drive toward lactate production after death.

Associated with the stress induced pathway is the detection of superoxide dismutase (SOD) which catalyses the conversion of the superoxide radical O_2_^–^ into oxygen or hydrogen peroxide. Along with glutathione peroxidase, these molecules represent the collective endogenously available first line of defence against free radicals [45]. SOD C and M increase significantly (*p* = 0.0001, 0.005. respectively) with PM time, and the increase in peroxide inactivation by SOD, is supported via increasing levels of glutathione peroxidase 1 and 3 to 720 h PM.

An alternative form of ATP production can occur via the recycling of ADP back to ATP by phosphate donation from phosphocreatine. The enzyme creatinine kinase controls this reaction and the membrane potential of H^+^ flux is managed by the voltage dependent anion transporter protein (VDAC) in the mitochondrial membrane [46]. Creatinine kinase (CK) and adenylate kinase are alternative processors to obtain energy within the cell. Over expression of CK has been associated with reduced apoptosis and poor prognosis for several tumours [47], with increased CK mRNA observed in senescence [48]. VDAC is the main gate keeper for the passage of anions, ATP, ADP and phosphates in its “open” state, while in the “closed” state it transports cations such as K^+^ and Ca^2+^ metabolites and nucleotides through the mitochondrial membrane. CK and the VDAC protein maintain the electrical potential across the mitochondrial membrane. These proteins, and additionally the adenine kinase protein, are increased through PM time (CKM *p* = 0.005, VDAC *p* = 0.0002) and are suggestive of an alternative form of energy homeostasis and stabilisation of the membrane potential long after death. Hexokinase is an anti-apoptotic protein that binds to VDAC to prevent cell injury by apoptosis and cell death; catalysing the conversion of glucose to substrates needed for oxidative phosphorylation and can be seen to be abundant with a maximum at 96 h PM.

### 3.3. Signalling Cascades in PM Time

The cellular stress response is also manipulated via post-translational modifications to support cellular integrity in an energy efficient manner [17]. An example of this is the post-translational phosphorylation of the Bcl2 protein which permits its continued translation from RBPs despite stresses that reduce the canonical forms of translation [20]. This results in the concomitant stabilisation of Bcl2 mRNA and resistance to apoptosis and the progression of cancer [20]. We demonstrate here, the passive regulation of signalling cascades involving HSP90. The HSP90 protein is an important regulator of at least 200 cellular proteins involved in multiple regulatory and signalling networks associated with cancer cell proliferation, survival and metastasis [49]. Its phosphorylation has been linked to the ability to successfully carry out chaperonin functions with phosphorylated HSP90 secretion being enhanced under oxidative stress conditions and aiding in the correct folding, stabilisation and refolding of denatured proteins [50]. HSP90 phosphorylation is coupled to its release from the target protein [51]. HSP90α and β were stably abundant in the proteomic profiles through PM time with HSP90β showing a rapid induction of the phosphorylated state with 2-fold increased levels of phosphorylated protein and concomitant decrease in unphosphorylated protein forms from 168 h. It is likely that at this time point PM, the constitutively phosphorylated protein can no longer regenerate to maintain its chaperonin function.

### 3.4. Disease Activation Pathways and Functional Enrichment

Evaluation of this dataset highlights the overriding element of cellular stress increasingly apparent through PM time. The stress response seen here, as with many other pathological conditions in the living proteome, is invoked through common environmental and physiological triggers. Cellular stress pushes cells into cell-cycle arrest, a beneficial mechanism that limits tumorigenesis, tissue damage and allows for the embryonic development of tissues, wound healing and pro-survival phenotypes. Long term senescence, however, paradoxically favours the development of pathologies such as cancer, heart disease and neuropathies, which have also been noted in this study and others [13,52]. The UPR and DDR are major drivers of senescence.

The UPR has been shown to influence cellular metabolism through endoplasmic reticulum (ER) Ca^2+^ signalling and can affect calcium and lipid transfer, and inhibition of protein translation via eIF2 phosphorylation, while calnexin and calreticulin trap partially folded proteins in the ER by binding to glycosylated proteins. Transduction of extracellular cues can affect local translation of the eukaryotic initiation factors which mediate the binding of tRNA_i_^Met^ onto the ribosome in a GTP dependent process. Terminally misfolded proteins are degraded through retro-translocation and the ubiquitin-proteasome system by hyperubiquitination or autophagy [53]. Additionally, extensive DNA damage causes programmed cell death with persistent damage triggering senescence. On a molecular level, the DDR allows promiscuous gene expression and promotes tumorigenesis. Induction of permanent cell-cycle arrest via increases in ROS is known as stress induced premature senescence (SIPS) and facilitates the secretion of pro-inflammatory cytokines, growth factors and proteases [54].

### 3.5. Commonalities between the “Death” Proteome and the Stress-Induced Senescence Phenotype

Proteins that contribute to the PM proteome are also considered in the senescence phenotype. In common to the SIPS phenotype are the expression of 133 proteins with regulatory functions stimulating transcription or acting as transcription factors; over 140 proteins sharing molecular functions of RNA binding, and a further eight proteins associated with the negative regulation of DNA methylation. Further commonality specific to the SIPS phenotype can be demonstrated in the increase of abundance of CAV1, IGFBP, ribosomal, apolipoproteins, EF1, 14-3-3 protein, S100, TYB4, H2AX, ARPC and ADAT proteins, and the sustained presence of LRP1, TGFβ, TFR1, HP1B3, interleukins and HSP90 across the PM proteome. Similarly, these are seen as changes in differential display and low-density DNA arrays studies [55,56]. Many of the oncogene-induced pathways common to oncogene-induced senescence (OIS) [57] including increased RAS are also abundant in the PM proteome. Together, the enrichment of pathological states and glycolytic pathways in addition to the tracking of protein complexes and individual proteins across PM timescales show a remarkable likeness to SIPS. Understanding how this relates to the expressed genetic content present in prehistoric human remains, as well as the numerous pathologies which invoke this phenotype will hold keystone indications to deciphering diseases of the past and present.

In conclusion, the death of an organism appears to have no immediate impact at the level of individual cells. It seemingly holds consequences only by way of the loss of oxygen which deprives cells of oxidative phosphorylation, the driver of ATP production and the main cellular energy currency. Redirection of nutrient availability via fat metabolism as well as increases in the glycolytic and TCA cycle enzymes contributes to diversion of the energy store to alternative forms of energy well into PM time scales. This is assisted by transcripts that can manipulate mRNA and enable protein synthesis and promiscuous gene-expression to continue. We demonstrated the increase and decrease of protein abundances across measurements of numerous mice through PM time. Normalisation of profiles against the 1 h-time point and validation of stable protein levels across the samples using collagen (a longer-lived protein) by immunoblotting confirm the uniform measurement of relative abundance through PM time. Heightened developmental pathways, cardiac dysfunction, neuropathies and carcinoma pathways enriched in the death transitioning of cells are the same pathways triggered in ageing and senescent phenotypes with increased significance through PM time. We have demonstrated that these pathways are held in the proteomic “memory” long after death. Characterisation of recently deceased PM proteomes is essential to further elucidate the data potential held within ancient proteomes as well as to inform on diseases of ageing into the future.

## 4. Materials and Methods

### 4.1. Experimental Design and Statistical Rationale

The mandible was chosen to be representative of a sample with the tensile and compressive strength as well as the protein-binding capacity within the protective bone matrix required to sustain preservation through deep-time [58]. Teeth, jaw and skull are often the only fragments remaining, able to directly preserve the life and death story of individuals in pre-history.

Wildtype male CBB6F1 mice were bred at the Australian Bio-Resources (ABR) in NSW Australia and transported and housed in cages with ad libitum access to food and water. Experiments were carried out with prior approval of the UNSW Animal Care and Ethics Committee (17/105A, 23 August 2017–22 August 2020) under the shared biological resources agreement, operating under animal ethics guidelines from the National Health and Medical Research Council (NHMRC) of Australia. Tissue from these mice was a shared resource. Twelve-week old (adult) mice with no underlying health conditions were euthanised by cervical dislocation and sample labelled “1” represents the left dentary which was extracted rapidly (completed within 1 h PM). Whole mouse was then reposed in a petri dish in a controlled 21 °C environment with unidirectionally controlled airflow in an open box until the collection of the right dentary at the next time point. Three dentaries from different mice were extracted at each time point of 1, 3, 6, 96, 168 and 720 h PM. We extracted 4 dentaries for the 24 h time point from 3 different mice. Twelve mice were used in total for this study. The power of the study was calculated to be 89% based on 7 time points and expression differences in 50% of the proteins of at least 1.5 fold using a multiple-treatment design [59].

Scaffold Software (version 4.6.1, Proteome Software Inc., Portland, OR, USA) was used to compare the mouse proteome through PM time using spectral counting. Peptide identifications were accepted if they could be established at greater than 95% probability using the Scaffold delta-mass correction. Protein identifications were accepted if they could be established at less than 1% false discovery rate (FDR) and contained at least 2 identified peptides. Expression changes across the samples were measured via spectral count, normalised by total ion count with missing values kept at zero and recorded as not detected (ND) during further analysis [60,61]. ANOVA was used to report abundance changes controlled by the Benjamini-Hochberg procedure for multiple comparisons, *p* values set to <0.05. Hierarchical clustering on z-scores normalised log2-transformed intensities was achieved using Perseus software in combination with MaxQuant software output [62] using the following criteria: 2 peptide minimum identification, protein present in at least 2 of 3 mice for each time point applying Euclidean distance. The proteomic dataset of differentially abundant proteins was assessed for enriched pathways using Ingenuity Pathway Analysis (IPA) (Qiagen, Redwood City, CA, USA). The core analysis was carried out using the default settings except that only direct relationships were considered based on the IPA knowledge base (genes only), with the stringent criteria of only experimentally observed confidence recorded.

### 4.2. Sample Preparation

Dentaries were prepared on ice by sonicating in a 1% solution of non-ionic detergent for 5 min, the removal of sinews by fine grade sandpaper and rinsing in icy sterile deionised water. Each dentary was weighed with a mean of 0.060 g (standard deviation 0.007 g, standard error 0.002 g and insignificant differences observed across PM time). The frozen dentaries were then crushed and washed for 18 h in 100 µL 0.5 M HCl at 4 °C on a shaker. Each sample was then washed in sterile deionised water, centrifuged at 14,000× *g*, and the pellet retained. Samples were resuspended in 50 µL AMBIC, 10 mM DTT, 2 M urea at pH 8 and 100 µg of total protein used for trypsin digestion at 25 °C for 16 h in a 1:100 ratio based on the weight of the extracted dentary. Digestion was halted by acidification.

### 4.3. Mass Spectrometry of Samples

Digested peptides were reconstituted in 5 μL 0.1% formic acid and separated by nano-LC using an Ultimate 3000 HPLC and autosampler (Dionex, Amsterdam, Netherlands) and followed methods similar to those described previously [58]. Briefly, the sample, 1.6 μg (1.7 μL from 10μL), was loaded onto a virgin micro C18 pre-column (300 μm × 5 mm, Dionex) with H_2_O:CH_3_CN (98:2, 0.1% TFA) at 10 μL min^−1^. After washing, the pre-column was switched (Valco 10 port valve, Dionex) into line with a virgin fritless nanocolumn (75 μm i.d × 20 cm) containing reverse phase C18 media (1.9 μm, 120 A, Dr. Maisch HPLC GmbH). Peptides were eluted using a linear gradient of H_2_O:CH_3_CN (98:2, 0.1% formic acid) to H_2_O:CH_3_CN (64:36, 0.1% formic acid) at 250 nL min^−1^ over 120 min. The QExactive (Thermo Electron, Bremen, Germany) mass spectrometer was run in DDA mode where a high voltage of 2000 V was applied to a low volume union and the column (45 °C) positioned 0.5 cm from the heated capillary (275 °C). A survey scan 350–1750 m/z was acquired in the Orbitrap (resolution 70,000 at 200 m/z) with an accumulation target of 10^6^ ions, lock mass enabled and up to the 10 most abundant ions (AGC target set to 10^5^, minimum AGC target set to 1.5 × 10^4^) with charge states ≥ +2 and ≤ +6 sequentially isolated and fragmented.

### 4.4. Protein Identification Relative Quantitation

Protein dataset-peak lists were generated from raw files using Mascot Daemon v2.5.1 (Matrix Science, London, UK, www.matrixscience.com). All MS/MS spectra were searched against the Uniprot database (downloaded Feb 2018); 556,568 sequences for protein identification with the following criteria: (1) taxon, Rodentia; (2) allowed 2 missed cleavages; (3) variable modifications, oxidation (M), phosphorylation (S,T,Y); (4) peptide tolerance, ±4 ppm; (5) fragment tolerance, ±0.4 Da; (6) peptide charge +2 and +3; and (7) enzyme specificity, semi-tryptic. A decoy database search was also performed. Only proteins identified with an adjusted false discovery rate (FDR) of 1%, with two unique peptides were used in further analysis.

### 4.5. Immunoblotting

Equivalent by weight portions of dentaries from the same mice used for label-free proteomic analysis were reserved for immunoblotting. Samples were resuspended in 100 µL of SDS buffer (NuSep, Sydney, Australia) and 15 µL was loaded onto nitrocellulose membrane using a slot blot apparatus. Membranes were blocked (10% milk powder, PBS) at 4 °C, followed by incubation overnight at 4 °C with one of the following antibodies: 1:700 dilution of Col1A2; 1:700 PGM1; 1:1000 DDX17; 1:2000 hnRNPF (Invitrogen, California, USA; PA5-51246, PA5-79382, PA5-41913, PA5-79382, respectively); or 1:10,000 EF2 (Abcam, Cambridge, UK; ab75748). The membranes were washed prior to incubation with goat anti-rabbit conjugated with HRP for 1 h, washed in chemiluminescence buffer, and immersed in SuperSignal West Femto (Thermofisher, Waltham, MA, USA) working solution for 5 min. Images were then captured using the LAS4000 system for chemiluminescence detection (GE Healthcare, Chicago, IL, USA).

### 4.6. PGM Colourimetric Activity Assay

Phosphoglucomutase assay (Abcam, Cambridge, UK; ab155896) was used to measure PGM conversion of glucose-1-phosphate to glucose-6-phosphate to form NADH which produces a coloured product with absorbance at 450 nm. The reported sensitivity of the kit is 1 mU/reaction. Activity was measured at 3 h and 168 h PM (time points displaying lower and higher levels of protein abundance in MS and immunoblot results). Standard curve with 0–20 nM NADH per well were prepared along with 3- and 168-h PM mouse mandible in duplicate, background mandible (without substrate added) and positive control as per manufacturer’s instructions. Activity was calculated from delta absorbance between 20 min and 60 min readings with the background subtracted.

## Supplementary Materials

The following are available online at https://www.mdpi.com/1422-0067/21/17/6422/s1.

## Abbreviations

PM	Post-Mortem
SIPS	Stress-induced premature senescence
RBP	ribosomal binding protein
