# Supplementary material for: The Molecular Floodgates of Stress-Induced Senescence Reveal Translation, Signalling and Protein Activity Central to the Post-Mortem Proteome
Source: Int J Mol Sci. 2020 Sep 3;21(17):6422. doi: 10.3390/ijms21176422 (PMC7504133; doi:10.3390/ijms21176422)
Supplement: Supplementary file 1 [file ijms-21-06422-s001.pdf]

Supplementary Materials

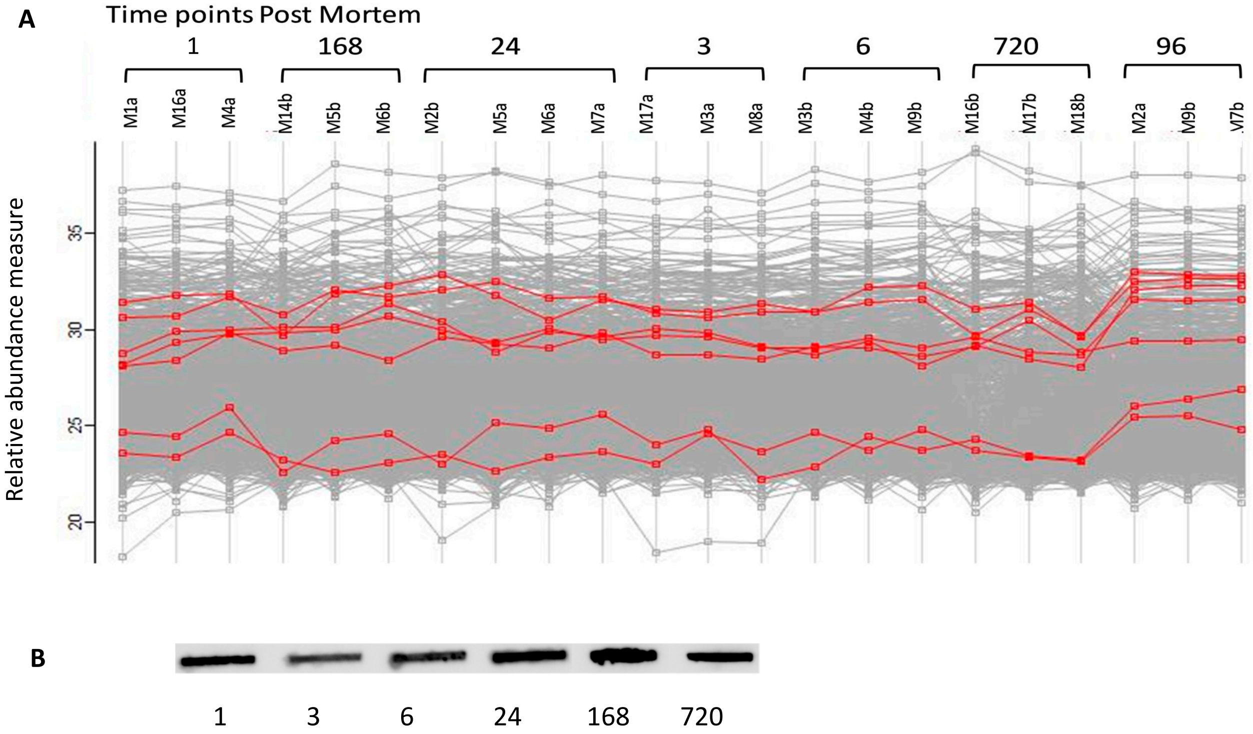

Figure S1. Normalisation in LC-MS experiments calibrate differences in samples based on sample amounts and corrected using a unique gain-factor for each sample based on a globally adjusted scale. Collagens are amongst the most abundant proteins in bone samples and have been demonstrated to be well preserved through time. Collagens do not undergo significant degradation over the time points assayed. (**A**) The average expression profile of 7 collagens are traced (red) against the background of all other mandible proteins (grey) across all samples assayed to show very consistent protein abundance profiles and insignificant deviations. This demonstrates the validity of using collagens as a 'sampling control' in this context to provide reliable relative quantitative data (ColA2: std dev 1.2, std err 0.48, and insignificant p-value across PM time). This reliability is confirmed using immunoblots (**B**) against COL1A2 antibody and demonstrates consistent levels of COL1A2 expression across 1, 3, 6, 24, 168, 720 PM time. The experiment was repeated twice with equivalent results.

Table S1. Changing dynamics of individual proteins representative of the Electron Transport Chain relative to 1 h time point. Not detected at these time points (ND).

	3	6	24	96	168	720
AT5F1	-1.1	-1	-1	1.1	-1.4	-2.4
ATP5E	-1.6	1.3	-1.5	-9.4	-10.5	ND
ATP5H	-1.4	-1.1	-1.4	-1.2	-1.8	-6.5
ATP5I	-1.2	1.1	-1.2	1.2	-1.7	-3.2
ATP5J	-1.2	1.2	-1.4	-2.3	-4.7	-11.2
ATP5L	-1	1	-1.4	-3	-4.4	ND
ATP8	-1.1	-1.1	-2	-17.5	-19.5	ND
ATPA	-1.2	-1.1	1	1.1	-1.3	-2.8
ATPB	-1	-1.1	-1.1	1.2	1.1	-2.4
ATPD	-2.3	-1.2	-1.3	1.1	-1.2	-7.2
ATPG	-2.1	1.3	-1	1	-1.2	-1.9
ATPK	1.5	1.6	1.2	-1.6	-3.5	ND
ATPO	-1.5	1.1	-1.1	-1.1	-1.9	-5.1
BLVRB	1.2	1.2	1.5	1.1	1.4	2
C560	1.1	1.2	2	4	2.7	ND
COX2	1.3	-1.4	2.1	1.7	1.8	-1.9
COX41	-1.7	-1.1	-1	-2.3	-2.7	ND
COX5A	-1.2	-1.1	-1.3	-1.1	-1.8	-5.5
COX5B	-1.7	-1	1.1	-1.3	-1.8	ND
COX6C	-1.9	-1.1	-1.5	-1.2	-3.3	-5.6
COX7C	-1.1	1.3	1.1	-1	-6	-2.4
COX8A	1.1	-2.6	-1.5	ND	ND	ND
CX6A1	-1.2	-1.6	-1.2	1.1	-2	-16.6
CX6A2	-2	-1.1	-2.1	ND	ND	ND
CX7A2	1.1	-2.7	-1.5	-1.2	-1.4	ND
CY1	-4.7	-1.6	1.9	-3.9	-1.3	ND
CYB5	-1.2	1.3	-1.1	-2.2	-9	ND
ETFA	-1.8	-1.3	-1.4	-1.6	-1.3	-1.9
FUMH	-1.8	-1.2	-1.5	-1	3.1	2.3
NB5R3	-1.9	-1.2	-1.3	-2.3	-2.4	-6.8
NDUA2	-2.4	-1.1	-1.3	-1.2	-3	ND

	3	6	24	96	168	720
NDUA3	-3.1	-9.8	-5.6	ND	ND	ND
NDUA4	-1.9	1.4	1.1	ND	ND	ND
NDUA5	-3.6	-1.5	-1.4	1.1	-2.9	ND
NDUA6	-1.3	1.1	-1.5	2.8	1.1	ND
NDUA7	-1.8	-1.1	-1.4	-1.4	-2.6	ND
NDUA8	-1.4	-1.3	-2.4	-6.8	-4.9	ND
NDUA9	-3.6	1.2	-1.3	1.8	1.6	ND
NDUAA	1.1	-1.8	1.4	1.6	-1.3	-3.9
NDUAC	1.1	1	1	-6.9	-8	ND
NDUAD	1.1	-1.8	1.6	-3.1	-1	ND
NDUB1	-5.8	-1.9	-2.1	ND	-5.4	ND
NDUB3	1.1	1.3	1	-1.4	-3.1	-4.8
NDUB4	-1.5	1.1	1	-6.3	-7.3	ND
NDUB5	-1.3	-2.1	-1.4	-5.4	ND	ND
NDUB6	-1.5	-1.3	-1.3	ND	-9.8	ND
NDUB7	1.5	1.5	1	1.1	-1.3	ND
NDUB9	1.2	-1	1	-1.5	-2.1	-3.4
NDUBA	-1	1.1	1.2	1.1	-3.2	ND
NDUBB	-1.2	-3.7	-1.6	-1.3	-7.4	ND
NDUC2	-1.2	-1.2	-1.2	ND	ND	ND
NDUS1	-1.6	-1.5	2.2	1.9	1	-3.6
NDUS3	-5.3	-5.9	-1.6	-1.6	1.4	-5.7
NDUS4	-2	-1	-1.8	1.5	-1.8	ND
NDUS5	-1.5	-1.1	-1.9	ND	ND	ND
NDUS6	-1.7	-1.3	-2	-1.7	-2.4	ND
NDUS7	-1.6	-1.9	-1.3	-1	-3.2	ND
NDUS8	-1.2	-1.3	1.1	-1.6	-2	ND
NDUV1	-6.4	-1.4	1.6	1.2	-1.2	-7
NDUV2	1	-1.3	1.1	1.1	-1.3	-12.4
NDUV3	ND	-1.3	1.1	1.6	-1.1	ND
NU5M	-1.4	2.1	-1.4	ND	ND	ND

	3	6	24	96	168	720
QCR1	-1.5	-1.1	1	1.3	-1.1	-12.9
QCR2	-1.6	-1.1	-1.1	1.3	-1.3	-8.3
QCR7	-2	1.2	-1.1	1.3	-1.2	-5.6
QCR8	-1.5	-1.1	-1.3	-1.4	-1.6	-2.2
QCR9	-1.3	-1	-1	-3.6	ND	ND
UCRI	-1	-1	-1.7	-1.1	-1.1	ND

Table S2. Changing dynamics of individual proteins representative of the ubiquitin proteasome relative to the 1 h time point.

	3	6	24	96	168	720
UBA1	1.6	-1.6	1.3	1.4	2.8	2.0
PSA1	-1.2	-2.1	-2.5	-5.9	-1.0	-1.6
UBE2N	-1.5	-1.3	-1.3	-6.5	-1.6	1.1
PSA2	-1.6	-3.5	-1.5	-1.1	2.7	2.3
PSMD2	3.4	1.7	4.1	2.6	2.7	-1.8
UB2L3	3.2	1.6	2.4	1.8	3.5	3.9
PSA7	2.8	1.1	1.6	1.3	3.4	6.4
UCHL1	-2.6	-3.2	-5.4	ND	ND	-2.4
UBE2O	-1.8	1.1	-1.9	ND	ND	ND
RS27A	-2.2	-2.3	-2.5	-2.9	-6.4	-5.2
PSB4	2.2	ND	2.8	1.3	5.5	2.5
PSA6	2.2	1.2	2.9	ND	-1.1	6.8
UBP5	ND	ND	-2.8	ND	-1.1	ND
ADRM1	-4.8	-2.5	-5.6	ND	-5.8	ND
UFM1	ND	ND	-1.1	ND	-1.1	2.1

Table S4: Changing dynamics of proteins involved with alternative energy production relative to the 1 h time point.

Table S3. Changing dynamics of Individual proteins representative of CytochromeP450 relative to the 1 h time point.

The post-mortem proteome is akin to stress-induced premature senescence.

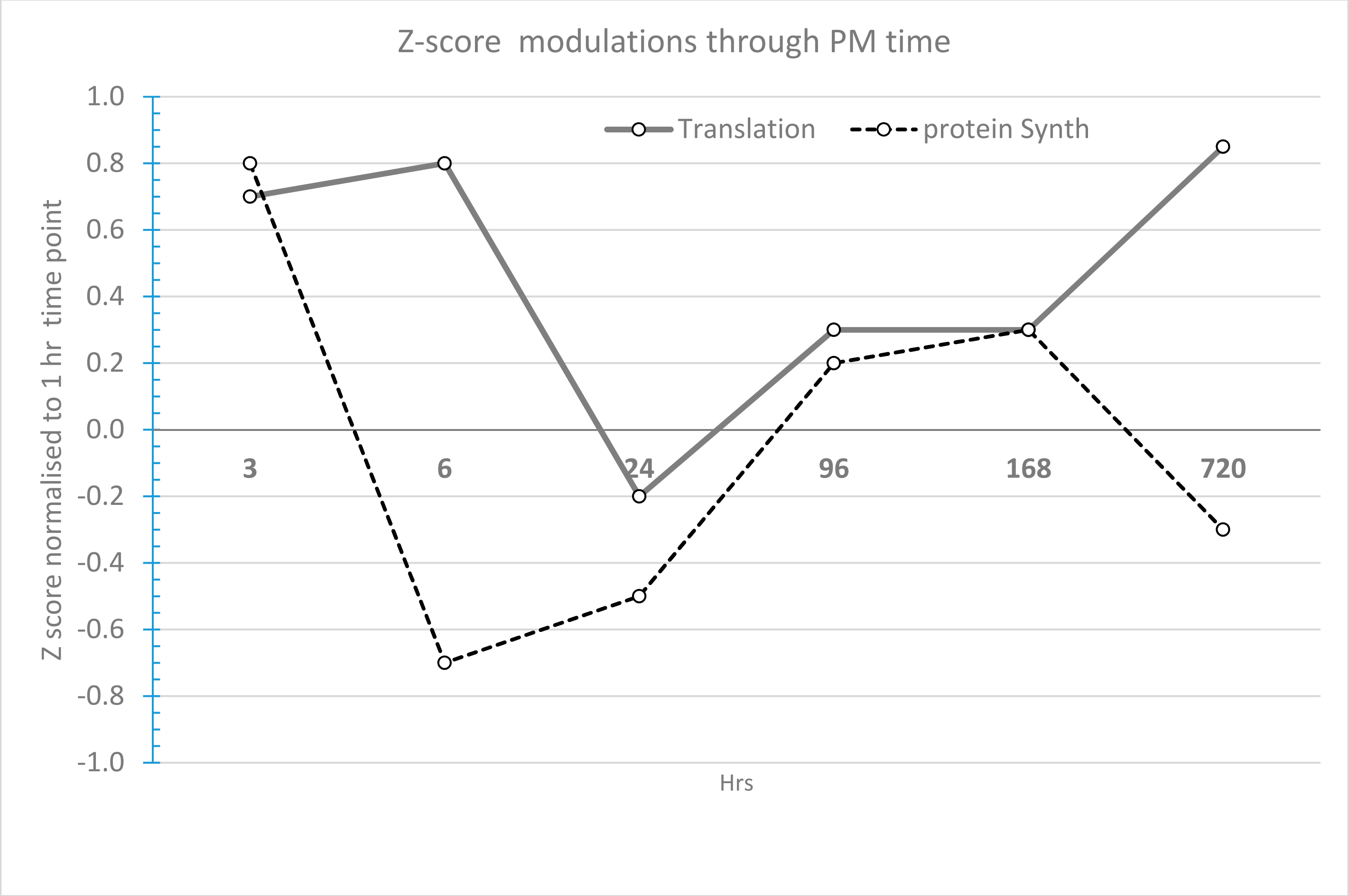

Figure S2. Z-score modulation through PM time for translation and protein synthesis. A z-score > |2| is representative of significance, while a z-score of < |1| represents no significant change.

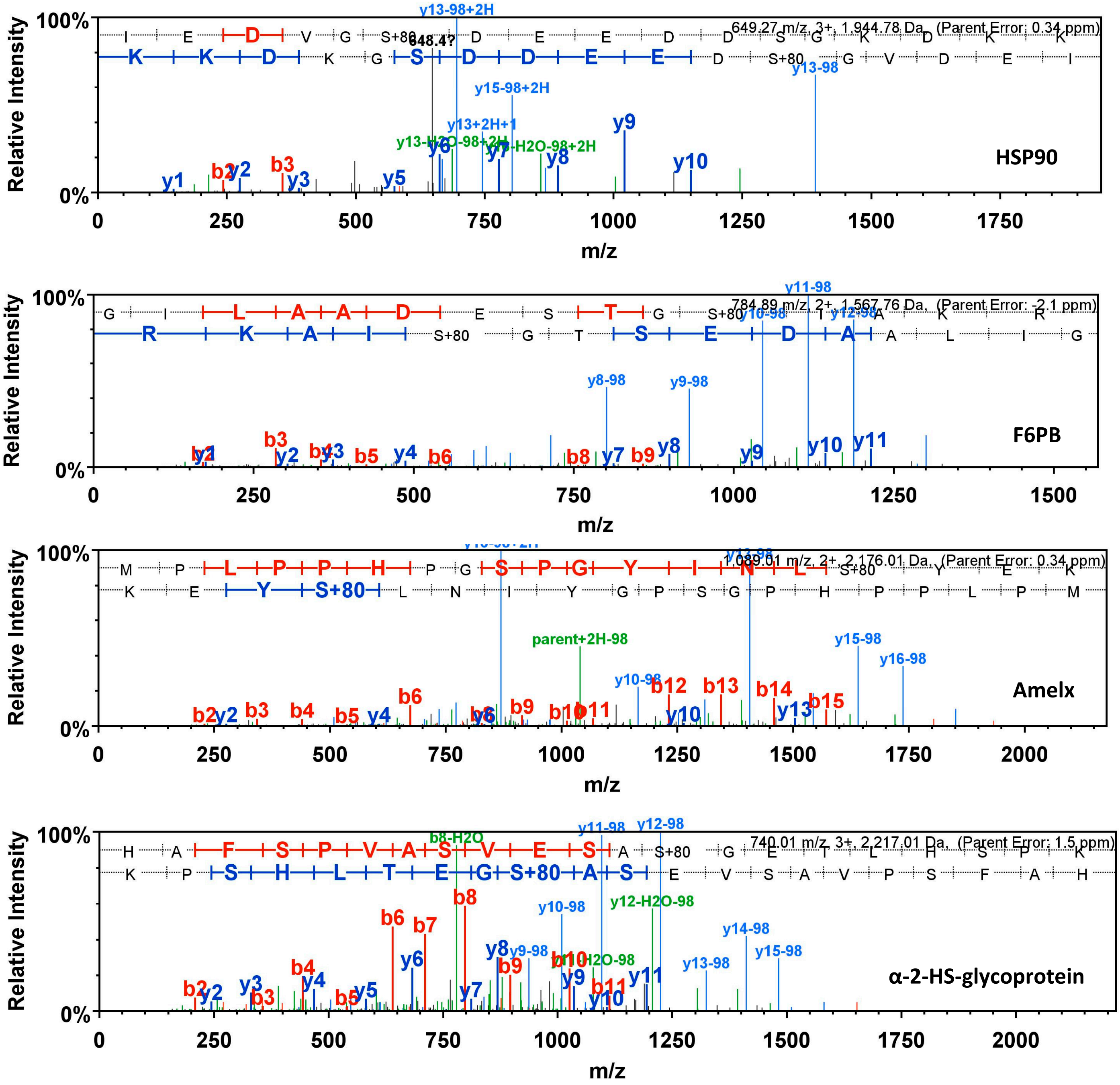

Figure S3. Phosphorylated peptide spectrum of HSP90, AmelX, α-2-HS-glycoprotein, and F6BP.
